# Simulation-based activated clotting time targeting for cardiopulmonary bypass in patients with antiphospholipid syndrome: two case reports

**DOI:** 10.1186/s44215-025-00235-0

**Published:** 2025-11-27

**Authors:** Yuki Kamikawa, Yosuke Saito, Hiromi Fujii, Yoichiro Machida, Hiroaki Hata, Hirotaka Inaba

**Affiliations:** 1 Department of Cardiovascular Surgery, Toda Chuo General Hospital, Saitama, Japan; 2 Department of Cardiovascular Surgery, Juntendo University Urayasu Hospital, 2-1-1 Tomioka, Urayasu, Chiba 279-0021 Japan

**Keywords:** Antiphospholipid syndrome, Activated clotting time, Cardiopulmonary bypass, Heparin monitoring, Aortic valve replacement, Simulation-based anticoagulation

## Abstract

**Background:**

Antiphospholipid syndrome (APS) is an autoimmune prothrombotic disorder that is frequently associated with systemic lupus erythematosus (SLE). Cardiac surgery in patients with APS presents unique challenges because activated clotting time (ACT) monitoring can be unreliable, frequently yielding falsely prolonged results due to the presence of lupus anticoagulant. Although heparin concentration-based monitoring is more accurate, devices such as the Hemostasis Management System Plus have been discontinued, creating a gap in practical anticoagulation management.

**Case presentation:**

We report two female patients with APS and severe aortic regurgitation (AR) who underwent aortic valve replacement (AVR) under cardiopulmonary bypass (CPB).

Case 1: A 58-year-old woman with long-standing SLE and APS underwent preoperative ACT simulation using her serum mixed with a 60 U/mL heparin solution to estimate the ACT corresponding to a heparin concentration of 3.0 U/mL. The resulting ACT range (567–708 s) guided intraoperative anticoagulation. AVR with a mechanical valve and left atrial appendage amputation (LAAA) was performed. Protamine was administered at half the calculated dose. Postoperative bleeding was transient and well controlled.

Case 2: A 62-year-old woman with recent APS, deep vein thrombosis, and infective endocarditis underwent ACT simulation. The simulated ACT ranged from 325 to 413 s, and an intraoperative ACT target of > 450 s was established. AVR and LAAA were performed uneventfully with half dose protamine. No thrombotic or bleeding complications occurred.

**Discussion:**

These cases highlight the limitations of conventional ACT monitoring in patients with APS and demonstrate the utility of individualized preoperative ACT simulation as a practical alternative. This approach allowed safe anticoagulation management without requiring advanced equipment. Reduced protamine dosing likely minimized the risk of rebound hypercoagulability, a concern in APS. Notably, despite identical heparin concentrations, ACT responses varied among the patients, underscoring the need for personalized strategies.

**Conclusion:**

Preoperative ACT simulation using patient serum offers a practical, accessible, and individualized method for guiding anticoagulation therapy in patients with APS undergoing cardiac surgery. This technique may enhance perioperative safety in resource-limited settings, and warrants further validation in larger cohorts.

## Background

Antiphospholipid syndrome (APS) is an autoimmune disorder associated with a heightened risk of arterial and venous thrombosis and pregnancy-related complications such as recurrent miscarriage [1]. Nearly half of APS cases are linked to systemic lupus erythematosus (SLE) [2]. In patients with APS undergoing cardiac surgery requiring cardiopulmonary bypass (CPB), perioperative anticoagulation is essential, but challenging. A major challenge is the pseudo-prolongation of the activated clotting time (ACT) due to interference from lupus anticoagulant, which complicates accurate assessment of the heparin effect [3].

Heparin concentration-based monitoring (e.g., anti-Xa assays and heparin-protamine titration) is considered superior to ACT in patients [4]. However, the discontinuation of the widely used Hemostasis Management System (HMS) Plus (Medtronic, Minneapolis, MN, USA) has created a gap in practical options for anticoagulation monitoring [5, 6]. This report describes two APS cases in which individualized ACT targets were preoperatively established using simulated heparin concentrations to guide safe and effective intraoperative anticoagulation therapy during CPB.

## Case presentation

### Case 1

A 58-year-old woman was referred to our hospital with progressive chest discomfort. She had a medical history of SLE and APS, diagnosed at 24 years of age, with prior neuropsychiatric lupus, hypertension, and dyslipidemia. Medications included panaldine 200 mg and prednisolone 1 mg. Transthoracic echocardiography (TTE) revealed severe aortic regurgitation (AR) with a preserved left ventricular ejection fraction (LVEF 67%). Laboratory tests revealed a hemoglobin level of 9.0 g/dL, platelet count of 85,000/μL, and activated partial thromboplastin time (APTT) of 56 s. Chest radiography revealed cardiomegaly with a cardiothoracic ratio of 56%.

**Fig. 1:**
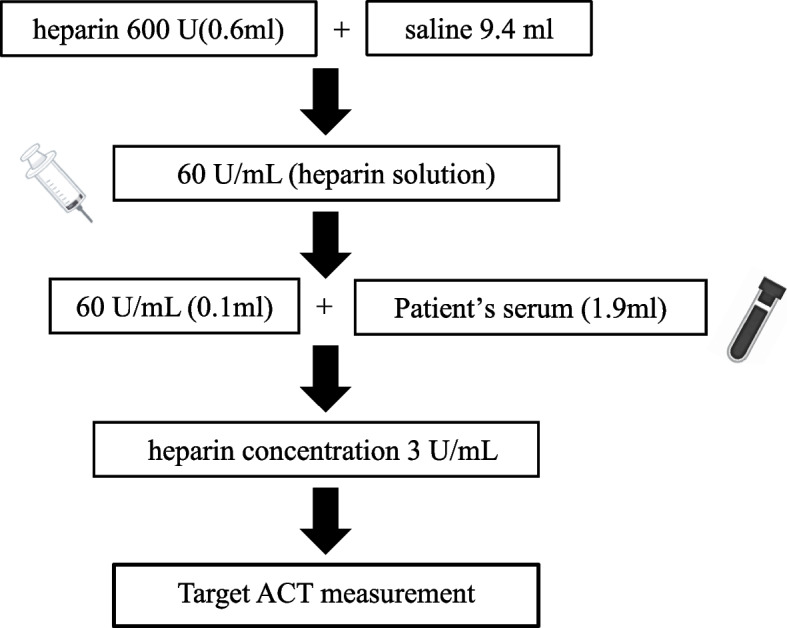
Simulated activated clotting time (ACT) corresponding to a heparin concentration of 3 U/mL. To establish an individualized anticoagulation target, patient serum was mixed with 0.1 mL of 60 U/mL heparin solution and 1.9 mL of serum to simulate a heparin concentration of approximately 3 U/mL. ACT was then measured to approximate the patient’s intraoperative anticoagulation response

We anchored the preoperative target ACT to a heparin concentration of 3.0 U/mL, which was chosen a priori as the minimum clinically safe target based on prior APS reports of heparin concentration–guided CPB demonstrating adequate anticoagulation at or above this level [4, 7, 8]. This anchor was used to define the individualized intraoperative target ACT for each patient (Fig. 1). ACT was measured using an Actalyke MINI II with Celite ACT Tubes (Helena Laboratories, Beaumont, USA). Heparin solution (60 U/mL) was added to the sample to simulate a plasma heparin concentration of 3.0 U/mL, resulting in ACT values of 708, 567, and 687 s. The target intraoperative ACT was set at 650–700 s.

**Fig. 2:**
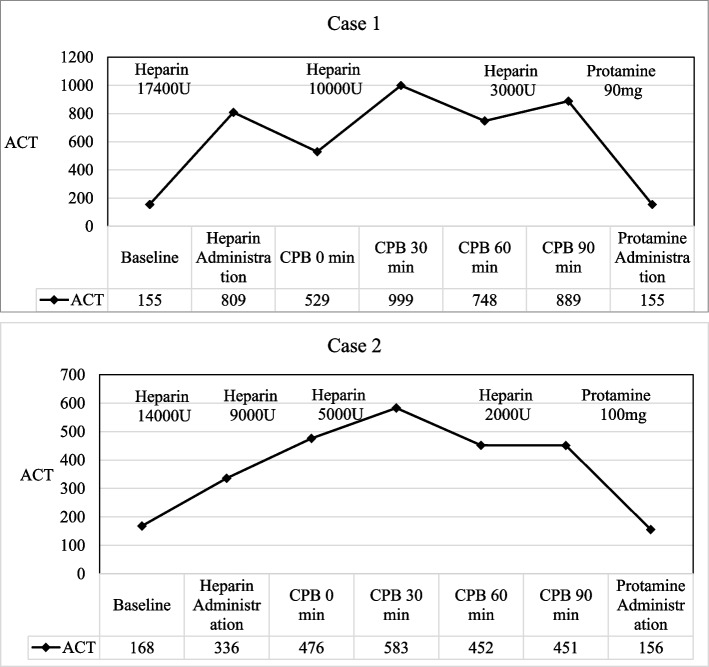
Intraoperative trends of activated clotting time (ACT), heparin dose, and protamine administration in Case 1 and Case 2. ACT values (left y-axis) and heparin/protamine doses are plotted over time. The graphs demonstrate that ACT targets determined by preoperative simulation were maintained throughout cardiopulmonary bypass. Half-dose protamine effectively reversed anticoagulation without complications

The patient underwent aortic valve replacement (AVR) with a mechanical valve of 21-mm and left atrial appendage amputation (LAAA) under CPB. The CPB circuit was prefilled with 5000 U of heparin. An initial bolus of heparin at 300 U/kg (total 17,400 U) increased the ACT to 809 s. Additional heparin infusions (10,000 and 3000 U) were administered intraoperatively to maintain the target ACT. At CPB termination, half of the calculated protamine dose (90 mg) was administered, returning the ACT to baseline (Fig. 2). The CPB, aortic cross-clamp, and total surgery times were 112, 87, and 230 min, respectively. Intraoperatively, 14 units of red blood cells (RBC), 8 units of fresh frozen plasma (FFP), and 20 units of platelet concentrate (PC) were required for hemostasis.

**Fig. 3:**
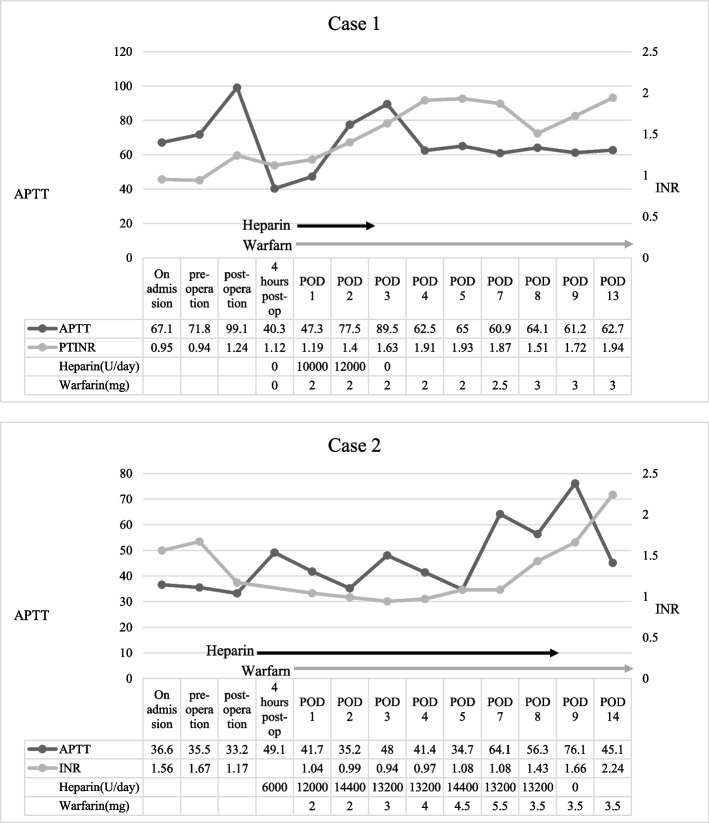
Postoperative anticoagulation and hemostatic parameters: Heparin timing and target (APTT), warfarin initiation and INR

Postoperative bleeding peaked 2 h after surgery, requiring transfusion of 6 units of RBC and 6 units of FFP. At the time of peak bleeding, the fibrinogen level was 188 mg/dl, and the INR was 1.24. FFP was administered to replace the coagulation factors, after which the drainage decreased and no re-exploration was required. Bleeding subsequently resolved without further intervention. Heparin was resumed on postoperative day (POD) 1, targeting an APTT of 1.5–2.0 multiplied by control, and warfarin was initiated on POD 1 with an INR goal of 2.0–3.0. The postoperative ACT values returned to baseline, and no thrombotic events occurred (Fig. 3). The patient was discharged on POD 9 with an uneventful postoperative course.

### Case 2

A 62-year-old woman with epigastric pain and oxygen desaturation was referred to our hospital. She had a medical history of SLE, APS, and deep vein thrombosis and was diagnosed at the age of 59 years. Prior symptoms included cellulitis, bleeding gastric ulcers, fever, and pyogenic spondylitis with vertebral fractures. The medications included aspirin 100 mg, warfarin 3 mg, and prednisolone 10 mg. TTE revealed a 5-mm vegetation on the noncoronary cusp of the aortic valve and severe AR, with a preserved LVEF of 75%. Laboratory tests on admission showed a hemoglobin level of 8.3 g/dL, platelet count of 367,000/μL, and APTT of 36.5 s. Chest radiography revealed a cardiothoracic ratio of 52.4%.

Given the potential unreliability of ACT for APS, a preoperative heparin titration test was performed using the same method as in Case 1 (Fig. 1). A 60 U/mL heparin solution was added to the sample to simulate a plasma heparin concentration of 3.0 U/mL, resulting in ACT values of 413, 325, and 398 s. An intraoperative ACT target of > 450 s was selected based on institutional standards.

The patient underwent AVR using a 19-mm mechanical valve and LAAA under CPB. A bolus of heparin at 300 U/kg (total 14,000 U) increased the ACT to 476 s. Additional heparin infusions (9000, 5000, and 2000 U) were administered to maintain adequate anticoagulation. At the conclusion of CPB, half of the calculated protamine dose (100 mg) was administered, successfully returning the ACT to baseline (Fig. 2). The CPB, aortic cross-clamp, and total surgery times were 147, 123, and 263 min, respectively. Intraoperatively, 14 units of RBC, 10 units of FFP, and 20 units of PC were required for hemostasis. Postoperative fibrinogen and INR were 212 and 1.17, respectively, with no postoperative bleeding complications. On POD 1, the patient received a transfusion of 2 units of RBC. Heparin was restarted on the same day with the same monitoring targets (APTT 1.5–2.0 multiplied by control), and warfarin was begun on POD 3 with an INR goal of 2.0–3.0. Postoperative ACT normalized without thrombotic complications (Fig. 3). The patient was discharged on POD 69 after receiving antibiotic therapy for infective endocarditis and underwent spinal fixation for pyogenic spondylitis.

## Discussion

Cardiac surgery in APS is challenging because perioperative thrombotic risk is high and inadequate anticoagulation during CPB contributes to adverse outcomes, with mortality reported up to 7% [3]. Heparin remains the intraoperative standard and is conventionally monitored by ACT. However, in APS, Lupus anticoagulants (LA) can pseudo-prolong ACT, decoupling it from the true anticoagulant effect and risking underdosing if ACT alone guides therapy [6, 9]. Ural and Edelson likewise advocate individualized strategies rather than reliance on ACT alone [5].

Heparin concentration-based approaches—anti-Xa assays and heparin–protamine titration (e.g., HMS Plus)—quantify heparin effect and show superior accuracy in APS and other coagulopathic states [4, 10]. Yet availability can be limited, and discontinuation of HMS Plus reduced practical point-of-care options. This gap underscores a need for methods that are both accessible and reliable.

We therefore used a preoperative calibration: patient serum was spiked to model a heparin concentration of 3.0 U/mL, the corresponding ACT was measured, and intraoperative heparin was titrated to that individualized ACT target. The ≥ 3.0 U/mL threshold was selected a priori from APS experience indicating that this level supports effective CPB anticoagulation [3, 7]. This strategy requires only standard ACT equipment and can be executed without specialized analyzers or on-site anti-Xa support.

In both cases, calibration yielded workable targets, and maintaining ACT within range was associated with stable anticoagulation without thrombosis or excessive bleeding. Simulated ACT values differed between the patients despite the same modeled heparin concentration, highlighting inter-individual variability in LA activity and baseline hemostasis. These findings support a patient-specific rather than one-size-fits-all approach when direct anti-Xa guidance is unavailable.

Protamine reversal warrants attention. Although full-dose protamine is guideline-directed for CPB reversal, it may precipitate rebound hypercoagulability in APS. We used half-dose protamine at separation from CPB, normalizing ACT without thrombosis or coagulopathy, supporting an initial conservative, titratable reversal guided by bleeding, drainage, and ACT trends [7, 8].

The relationship between ACT and heparin effect is imperfect—particularly at higher heparin concentrations and in LA-positive patients—and can be distorted by hypothermia, hemodilution, and perfusion factors during CPB. Our method is best regarded as calibration rather than quantification: anchoring intraoperative ACT to a predefined heparin concentration when anti-Xa assays are unavailable. Where anti-Xa monitoring is accessible, it should be preferred and, when feasible, paired with simulation-anchored ACT targets and clinical assessment.

This study has limitations. In-vitro calibration assumes a stable relationship between simulated and in-vivo ACT that can shift with intraoperative physiology. The method does not provide real-time heparin quantification and thus requires vigilant monitoring and judgment. Finally, we used a single concentration point (3.0 U/mL) as a minimum safe threshold rather than a full heparin–ACT curve (1.0–5.0 U/mL). Future work should prospectively evaluate multi-point titration with anti-Xa validation and include non-APS comparators to clarify in-vitro–in-vivo correlation and outcomes.

In conclusion, preoperative, patient-specific ACT calibration is a simple, low-cost way to individualize anticoagulation for APS during CPB when direct heparin assays are not readily available. In two APS patients undergoing AVR, calibration-anchored targets, conservative protamine reversal, and structured postoperative bridging enabled safe anticoagulation without thrombotic events. Prospective, multi-center studies with multi-point titration and anti-Xa validation are warranted to confirm reproducibility and generalizability.

## Data Availability

The datasets used and analysed during the current study are available from the corresponding author on reasonable request.

## Abbreviations

APS: Antiphospholipid antibody syndrome
SLE: Systemic lupus erythematosus
CPB: Cardiopulmonary bypass
ACT: Activated clotting time
HMS: Hemostasis Management System
TTE: Transthoracic echocardiography
AR: Aortic regurgitation
LVEF: Left ventricular ejection fraction
APTT: Activated partial thromboplastin time
AVR: Aortic valve replacement
LAAA: Left atrial appendage amputation
RBC: Red blood cell
FFP: Fresh frozen plasma
PC: Platelet concentrates
POD: Postoperative day
LA: Lupus anticoagulants
